# A Nonpalpable Nodule in Ectopic Axillary Breast Tissue: Consider Phyllodes Tumor

**DOI:** 10.1155/2016/3603262

**Published:** 2016-12-26

**Authors:** Eva Ruvalcaba-Limón, Verónica Bautista-Piña, Julio Ramírez-Bollas, Ruby Espejo-Fonseca, Sergio Rodríguez-Cuevas

**Affiliations:** ^1^Department of Breast Surgical Oncology, Instituto de Enfermedades de la Mama y Fundación del Cáncer de Mama (IEM-FUCAM), Mexico City, Mexico; ^2^Department of Pathology, IEM-FUCAM, Mexico City, Mexico; ^3^Department of Radiology, IEM-FUCAM, Mexico City, Mexico

## Abstract

Benign and malignant pathology can develop in ectopic axillary breast tissue, such as fibroadenomas, phyllodes tumors, and breast cancer. We present a rare case of an asymptomatic 43-year-old woman with an axillary nodule which was identified during screening mammography within ectopic axillary breast tissue, initially considered as a suspicious lymph node. Radiologic studies were considered as Breast Imaging-Reporting Data System (BI-RADS) 4. A hyperdense, lobular, and well-circumscribed nodule was identified in mammogram while the nodule by ultrasound (US) was hypoechoic with indistinct microlobular margins, without vascularity by Doppler, and measuring 1.26 × 1 cm. Core-needle biopsy reported a fibroepithelial neoplasm. The patient was submitted to local wide-needle excision located in intraoperative radiography of the surgical specimen and margin evaluation. Final histopathological study reported a 1.8 × 1.2 cm benign phyllodes tumor, with irregular, pushing, and clear wide margins within normal ectopic breast tissue. The patient without surgical complications continued annual screening without recurrence during a follow-up that took place 24 months later.

## 1. Introduction

Benign and malignant pathology can develop in breast tissue, even if the mammary tissue is localized in ectopic foci in the axillary area. Benign pathology can identify fibroadenomas [1, 2], as well as malignant disease such as breast cancer [3, 4]. The presence of nodules in axillary breast tissue is extremely rare, and they are usually confused with axillary lymph nodes.

## 2. Case Presentation

**Figure 1:**
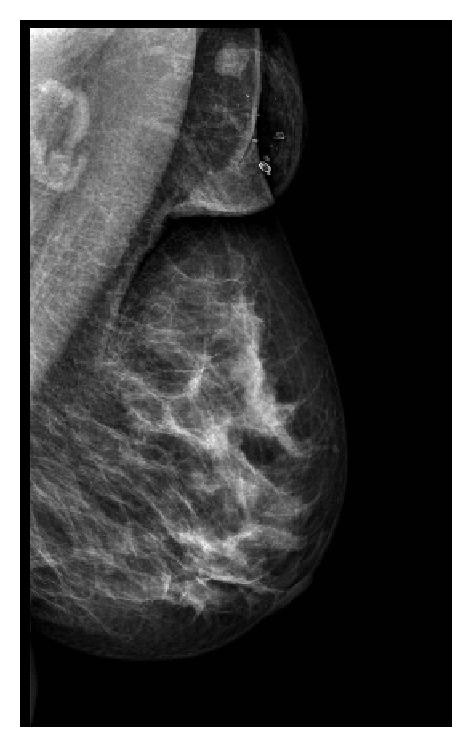
Nonpalpable hyperdense, lobular, and well-circumscribed nodule in ectopic axillary breast tissue in digital mammographic study.

**Figure 2:**
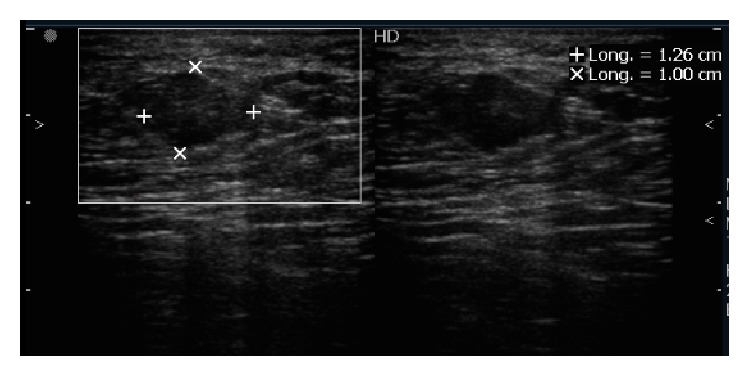
Hypoechoic nodule with indistinct microlobular margins and without vascularity in US study.

**Figure 3:**
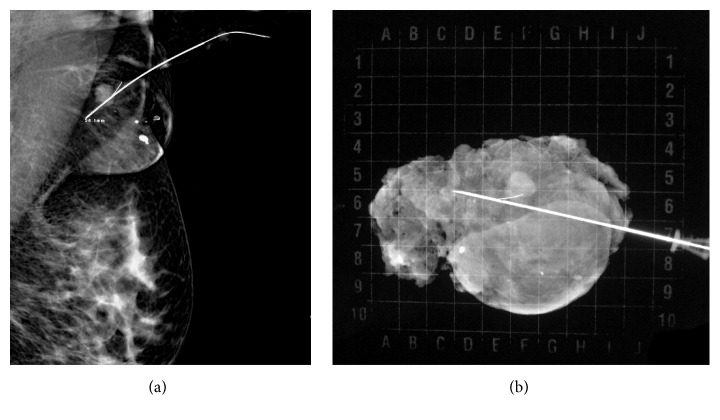
Wide-needle localized excision (a) with intraoperative radiography of surgical specimen (b).

**Figure 4:**
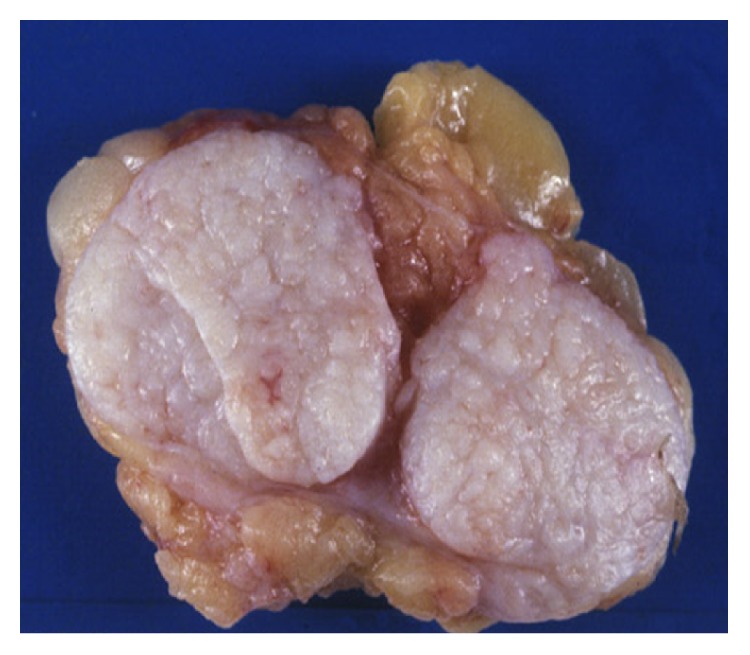
A 1.8 × 1.2 cm round multinodular soft mass, with well-circumscribed lobulated contours, focal with irregular borders.

**Figure 5:**
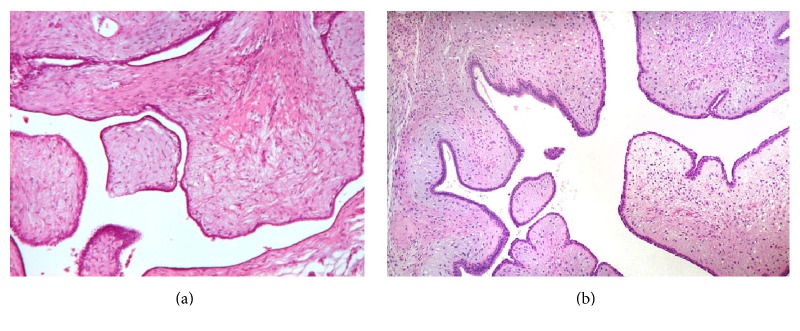
(a, b) H&E ×10 intracanalicular pattern forming cleft-like spaces; the stroma is mildly atypical with one mitosis per 10 high-power field.

This is a case of a 43 years old woman who had systemic high blood pressure, multiparous with three pregnancies and three vaginal deliveries, with positive breastfeeding after 1 year on each birth. She has not used hormone contraception; body mass index was 26.3 kg/m^2^, and there was bilateral ectopic axillary breast tissue that increased in volume after the pregnancies. At her first screening mammography at a mobile unit, a nodule was identified on the left axillary. Mammography findings were diagnosed as Breast Imaging-Reporting Data System (BI-RADS) 4 due to the presence of a hyperdense, lobular, and well-circumscribed nodule, suggested as a suspicious lymph node (Figure 1). By ultrasound (US) imaging, hypoechoic nodule measuring 1.26 × 1 cm was identified, with indistinct microlobular margins and without vascularity identified by Doppler (Figure 2). During physical exploration, there was only the presence of bilateral ectopic axillary breast tissue with a volume of 8 × 8 cm, without palpable lumps in the patient's mammary glands or in the ectopic tissue. The histopathological study from the US-guided core-needle biopsy revealed a fibroepithelial neoplasm. The patient underwent wide-needle localized excision with intraoperative radiography of surgical specimen (Figures 3(a) and 3(b)) and evaluation of tumor margins by the pathologist. The final histopathological study reported the presence of benign phyllodes tumor with clear wide margins (>10 mm) in a normal ectopic breast tissue. The gross description reported a round multinodular soft mass, 1.8 × 1.2 cm in dimension; tumor demonstrated a white cut surface and well-circumscribed lobulated contours, focal with irregular borders (Figure 4). The microscopic description in low-power views showed a fibroepithelial lesion with intracanalicular growth patterns that form cleft-like spaces, infiltrating focal borders, stromal heterogeneity with myxoid and hypercellular areas, mild cell atypia, and one mitosis per 10 high-power field. Epithelial and benign glandular elements with florid hyperplasia were present (Figures 5(a) and 5(b)). The patient without surgical complications continued annual screening without recurrence during a follow-up that took place 24 months later.

## 3. Discussion

Asymptomatic cases with phyllodes tumor of the breast is uncommon, and those that grow from ectopic breast tissue are extremely rare. Wherever the localization of ectopic breast tissue (axillary, inframammary, crude, and vulvar), it could develop any benign and/or malignant disease [5]. There are few cases of fibroepithelial neoplasm localized in axilla, such as fibroadenomas [1, 2] or less commonly phyllodes tumors [6–8]. Diagnosis should be performed with core-needle biopsy, and treatment with surgical excision with wide margins is mandatory.

To classify benign, borderline, or malignant phyllodes tumor, the pathologist needs to analyze the whole surgical specimen. Nonpalpable mammary lesions could be submitted to needle-guided excisional biopsy with intraoperative evaluation of the surgical specimen, as well as the three-dimensional margins to ensure wide margins [9]. Very small phyllodes tumors are reported in fewer than 10% [10, 11] but, in geographical settings with breast cancer screening programs, these could increase to 31% [12]. At our institute, asymptomatic phyllodes tumors were documented in 8.1% (25/307 cases) during a 10-year period, and only one case was localized in axillary tissue (0.3%).

The main differential diagnosis is fibroadenoma which is especially difficult on core biopsies. Parameters favoring phyllodes tumor diagnosis included increased stromal cellularity, pleomorphism, stromal overgrowth, and presence of mitoses. As in our case phyllodes tumor with infiltrating borders must be differentiated from periductal stromal sarcoma; the main histologic features is that the last one lacks a leaf-like growth pattern and is composed of multiple nodules separated by nonneoplastic tissue. Immunohistochemistry stains have limited value in differential diagnosis of fibroepithelial neoplasms; despite the research efforts, morphology remains the gold standard for the diagnosis of these tumors.

However, even this phyllodes tumor was in uncommon localization; diagnosis and treatment are similar to those in other nonpalpable phyllodes tumors in normal mammary gland.
